# Supplementary material for: Involvement of mutant and wild-type CYSLTR2 in the development and progression of uveal nevi and melanoma
Source: BMC Cancer. 2021 Feb 15;21:164. doi: 10.1186/s12885-021-07865-x (PMC7885466; doi:10.1186/s12885-021-07865-x)

# Supplementary information

## Supplementary Table 1

Context sequences, PCR annealing temperatures and supplier information for all assays used.

| Assay (supplier) | *GNAQ* p.Q209P/L mutation and wild-type (Bio-Rad Laboratories) |
| --- | --- |
| Context Sequence | AGTGTATCCA TTTTCTTCTC TCTGACCTTT GGCCCCCTAC ATCGACCATT  CTGCAAGGTT AACAATACTC ATATTAATAA CATATAAAGT AAAACTAAAA  AGTCAACATA AATATAGCAC TAC |
| Location (hg19) | chr9:80409459-80409581 |
| Amplicon length | 65nt |
| PCR annealing temperature | 55 °C |

| Assay (supplier) | *GNA11* p.Q209P/L mutation and wild-type (Bio-Rad Laboratories) |
| --- | --- |
| Context Sequence | CTTTCAGGAT GGTGGATGTG GGGGGCCAGC GGTCGGAGCG GAGGAAGTGG  ATCCACTGCT TTGAGAACGT GACATCCATC ATGTTTCTCG TCGCCCTCAG  CGAATACGAC CAAGTCCTGG TGG |
| Location (hg19) | chr19:3118917-3119037 |
| Amplicon length | 62nt |
| PCR annealing temperature | 55 °C |

| Assay (supplier) | *CYSLTR2* p.L129Q mutation and wild-type (Sigma-Aldrich) |
| --- | --- |
| Context Sequence | TCAGGGCTGA CTATTATCTT AGAGGCTCCA ATTGGATATT TGGAGACCTG  GCCTGCAGGA TTATGTCTTA TTCCTTGTAT GTCAACATGT ACAGCAGTAT  TTATTTCCTG ACCGTGCTGA GTGTTGTGCG TTTCCTGGCA ATGGTTCACC  CCTTTCGGCT TCTGCATGTC ACCAGCATCA |
| Location (hg19) | chr13:49281231-49281410 |
| Amplicon length | 101nt |
| PCR annealing temperature | 55 °C |

| Assay (supplier) | *SETDB2* rs2057413 variants chr. 13q (Sigma-Aldrich) |
| --- | --- |
| Context Sequence | AAACTGAGAA ATGTCCACCA AAGTTCAGTA ATAATCCCAA GGAGCTTACT  GTGTAAGTAA CAGCTGAGGA ACCCAGAGTA AATCTAAATT ATTATCAATC  AATTGGTTCT TTTTCA |
| Location (hg19) | chr13:50057047-50057162 |
| Amplicon length | 73nt |
| PCR annealing temperature | 55 °C |

| Assay (supplier) | *SPCS1* rs6617 variants chr. 3p (Sigma-Aldrich) |
| --- | --- |
| Context Sequence | TTCCGGGGCC GCCGCCATCG CTCTCCCGGG CTTAGAAGGC CCGGCTACTG ACGCGCAGTG CCAGACCTTA CCCCTCACGG TCCTTAAGTC TCGGTCGCCC TCGCCTCGCA GCCTGCCACC CGCGCTCAGC TGCCCGCCTC CTCAGCCAGC |
| Location (hg19) | chr3:52740112-52740261 |
| Amplicon length | 63nt |
| PCR annealing temperature | 55 °C |

| Assay (supplier) | *TERT* reference chr. 5p (Bio-Rad Laboratories) |
| --- | --- |
| Context Sequence | CACCCCTTGG TGGCGGCTCA CCTGTACGCC TGCAGCAGGA GGATCTTGTA  GATGTTGGTG CACACCGTCT GGAGGCTGTT CACCTAGAGT CGCCAAGAAA  GAGTGAGAAA CGGTAGAAAC CTC |
| Location (hg19) | chr5:1258692-1258814 |
| Amplicon length | 100nt |
| PCR annealing temperature | 55 °C / 60 °C |

| Assay (supplier) | *VOPP1* reference chr. 7p (Bio-Rad Laboratories) |
| --- | --- |
| Context Sequence | TATGGAGAGG GCCCGCACAC AGCACCTGGA GCCACAGCAG TCCTCGTAGG  AGCGGCATCT GTGGAGAGAG GCACAGGCTG GTCAGCACTG AATTGGAAGC  AGCCACCGGA CCAGCCATGC GGC |
| Location (hg19) | chr7:55565326-55565448 |
| Amplicon length | 64nt |
| PCR annealing temperature | 60 °C |

| Assay (supplier) | *TTC5* reference chr. 14q (Bio-Rad Laboratories) |
| --- | --- |
| Context Sequence | TGGTCGCGAT GCCACTGTGG CAACAGCCTG GCTGCTGGAT CCCTGAGGCT  TCCCATTCAC CACTAGCAGG AGGGGCGTCT CCACTCGAAC ACTGGAAAAG  GAATAGTCCT AGAAAAGACA GAC |
| Location (hg19) | chr14:20757798-20757920 |
| Amplicon length | 59nt |
| PCR annealing temperature | 55 °C / 60 °C |

| Assay (supplier) | *CDC42* target chr. 1p (Bio-Rad Laboratories) |
| --- | --- |
| Context Sequence | GGATACAAAA CTATTTCAGC AATGCAGACA ATTAAGTGTG TTGTTGTGGG  CGATGGTGCT GTTGGTAAAA CATGTCTCCT GATATCCTAC ACAACAAACA  AATTTCCATC GGAATATGTA CCG |
| Location (hg19) | chr1:22404951-22405073 |
| Amplicon length | 98nt |
| PCR annealing temperature | 60 °C |

| Assay (supplier) | *PTK2* target chr. 8q (Bio-Rad Laboratories) |
| --- | --- |
| Context Sequence | CAACCAGATG GTCATTCAAA AAAGTTGGAG CTGTAAGTGC TGGCGACTGA  GGACACAGGG TTAATTCCTC GCTGCTGGTG GAAGGCTAGA GAACATCTTC  AAAAGAGGGT AGCAAGACGT GCT |
| Location (hg19) | chr8:141669430-141669552 |
| Amplicon length | 70nt |
| PCR annealing temperature | 60 °C |

| Assay (supplier) | *NFAT5* target chr. 16q (Bio-Rad Laboratories) |
| --- | --- |
| Context Sequence | TGGAAGCCAT GAGAGAGAAT ATGATTTCAG GAAAACTTGG GCACAGAGAA  TTTGGCCTAT CTCAGTATGA CAAACTAATA GCTGTTGTGA TTCATTTAGG  GACTGTTGTT AAATATTTGA TTA |
| Location (hg19) | chr16:69715015-69715137 |
| Amplicon length | 69nt |
| PCR annealing temperature | 60 °C |

## Supplementary Data 1

Methodical overview of all digital PCR experimental setups.

Digital PCR experiments were carried out using the QX200™ Droplet Digital™ PCR System (Bio-Rad Laboratories, Hercules, USA) following the protocols and general guidelines described earlier (1-3). In short, 20 ng DNA was analysed in a 22 uL reaction volume, using 11 uL ddPCR™ Supermix for Probes (No dUTP, Bio-Rad) and primers and probes in final concentrations of 900 and 250 nM. MIQE context sequences and supplier information for all assays used are provided in **Supplementary Table 1**.

PCR mixtures were partitioned into 20.000 droplets using the AutoDG™ System (Bio-Rad Laboratories). Consequent PCR was performed in a T100 Thermal Cycler using the following protocol: 10 min at 95 °C; 30 s at 94 °C and 1 min at 55 °C or 60 °C (depending on the assay, see **Supplementary Table 1**) for 40 cycles; 10 min at 98 °C; cooling at 12 °C for up to 48 h, until droplet reading. Ramp rate was set to 2 °C/s for all steps. Reading of the droplets was performed using a QX200™ Droplet Reader (Bio-Rad Laboratories).

The *CYSLTR2* p.L129Q mutation was analysed in a duplex experiment using a FAM-labelled probe for the mutant, and HEX-labelled probe for the wild-type allele (**Figure 1**). Mutant allele fractions (MAF) were determined as follows:

$$\mathrm{MAF}=\frac{[\text{mutant}]}{\left[ \text{mutant} \right]+[\text{wildtype}]}$$

The MAF can be used to estimate the mutated cell fraction (MCF) under heterozygous conditions (i.e. nevus 12B):

$$\text{MCF}=2\cdot\text{MAF}$$

Chromosomal alterations were analysed in a duplex experiment using a FAM-labelled assay for the target and a HEX-labelled assay for the reference. Targets included *CDC42* (chromosome 1p), *PTK2* (chromosome 8q) and NFAT5 (chromosome 16q), references included *TERT* (chromosome 5p), *VOPP1* (chromosome 7p) and *TTC5* (chromosome 14q), as previously introduced (1, 2).

Copy number values (CNV) were determined as follows:

$$\text{CNV}=2\cdot\frac{[\text{target}]}{[\text{reference}]}$$

Although *TERT*, *VOPP1* and *TTC5* are located on chromosomes (5p, 7p and 14q, respectively) not frequently lost or gained in primary uveal melanoma (see also **Supplementary Figure 3)** (1, 4), individual tumours may contain copy number alterations involving these loci. To select a copy number invariant reference for each tumour, we compared the concentrations of *TERT*, *VOPP1* and *TTC5* with each other. Ratios between the references were calculated as follows:

$$\text{Ratio}=\frac{[\text{reference 1}]}{[\text{reference 2}]}$$

| **PUM-1A** |  | **PUM-2A** |
| --- | --- | --- |
| 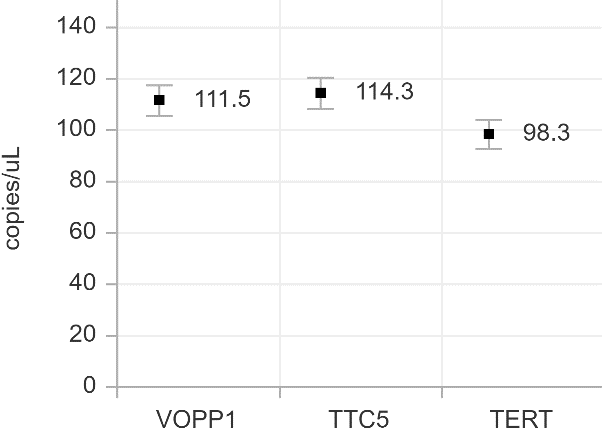 |  | 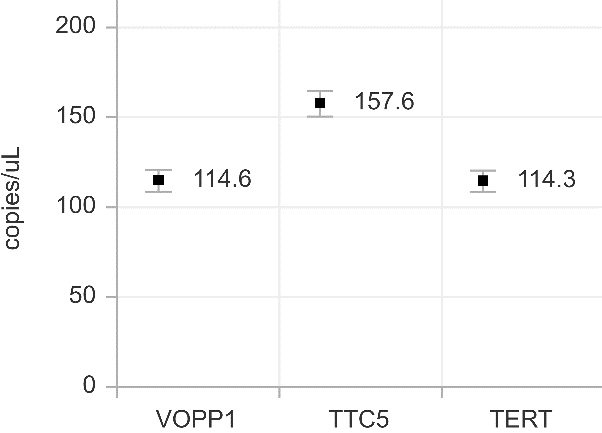 |
|  |  |  |
| 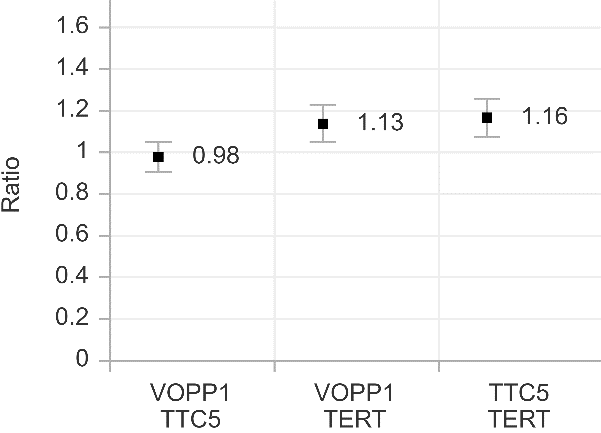 |  | 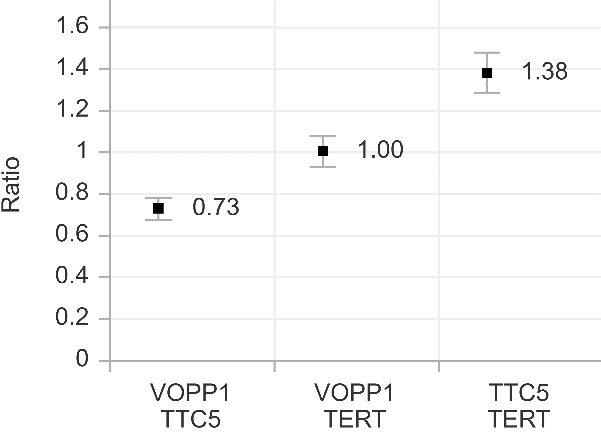 |

In PUM-1A, *VOPP1* and *TTC5* showed a similar concentration, resulting in a ratio [*VOPP1*]/[*TTC5*] almost equal to 1. However, *TERT* was significantly lower abundant. We therefore excluded *TERT* as potential reference and (arbitrarily) chose to use *TTC5* as stable reference for this tumour.

In PUM-2A, VOPP1 and TERT showed a very similar concentration, resulting in a ratio [*VOPP1*]/[*TERT*] equal to 1. However, *TTC5* was significantly higher abundant. We therefore excluded *TTC5* as potential reference and (arbitrarily) chose to use *TERT* as stable reference for this tumour.

Concerning the CNV of *CYSLTR2*, a multiplex experiment was carried out using a FAM-labelled assay for mutant *CYSLTR2*, and a HEX-labelled assay for the stable reference. This setup results in an extra ‘predictive cluster’ for *CYSLTR2* wild-type alleles, which is derived from suboptimal binding of the mutant probe to wild-type alleles in absence of wild-type probe. This suboptimal binding does not cause the wild-type alleles to be inadequately quantified, it only leads to a lower amplitude of the cluster droplets filled with wild-type alleles, compared to the cluster droplets filled with mutant alleles (2). The multiplex is completed with a cluster double-positive droplets, containing *CYSLTR2* mutant and wild-type alleles. Multiplexing in 2D digital PCR experiments has been described in detail by Whale et al. (5). The annotation and interpretation of our *CYSLTR2* multiplex setup is given below:

| **2D plot**  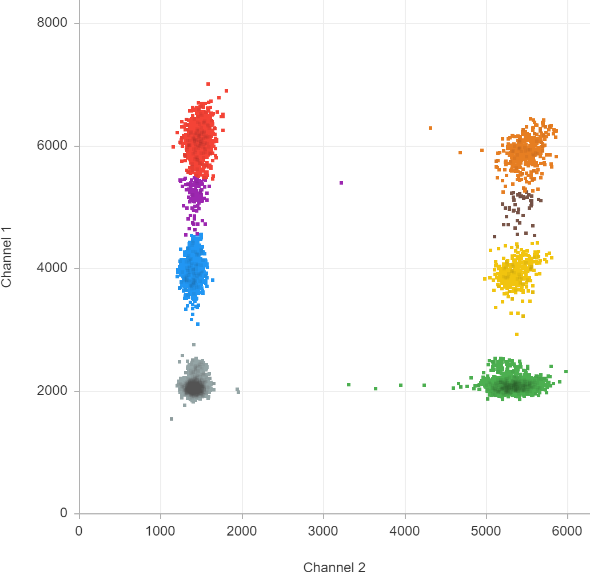 | \| **2D plot annotation** \| \| \| \| \| --- \| --- \| --- \| --- \| \| ⚫ \| *CYSLTR2* mutant+  *CYSLTR2* wild-type-  reference- \| ⚫ \| *CYSLTR2* mutant+  *CYSLTR2* wild-type-  reference+ \| \| ⚫ \| *CYSLTR2* mutant+  *CYSLTR2* wild-type+  reference- \| ⚫ \| *CYSLTR2* mutant+  *CYSLTR2* wild-type+  reference+ \| \| ⚫ \| *CYSLTR2* mutant-  *CYSLTR2* wild-type+  reference- \| ⚫ \| *CYSLTR2* mutant-  *CYSLTR2* wild-type+  reference+ \| \| ⚫ \| *CYSLTR2* mutant-  *CYSLTR2* wild-type-  reference- \| ⚫ \| *CYSLTR2* mutant-  *CYSLTR2* wild-type-  reference+ \| |
| --- | --- | --- | --- | --- | --- | --- | --- | --- | --- | --- | --- | --- | --- | --- | --- | --- | --- | --- | --- | --- | --- |
|  |  |

**Absolute quantification and interpretation**


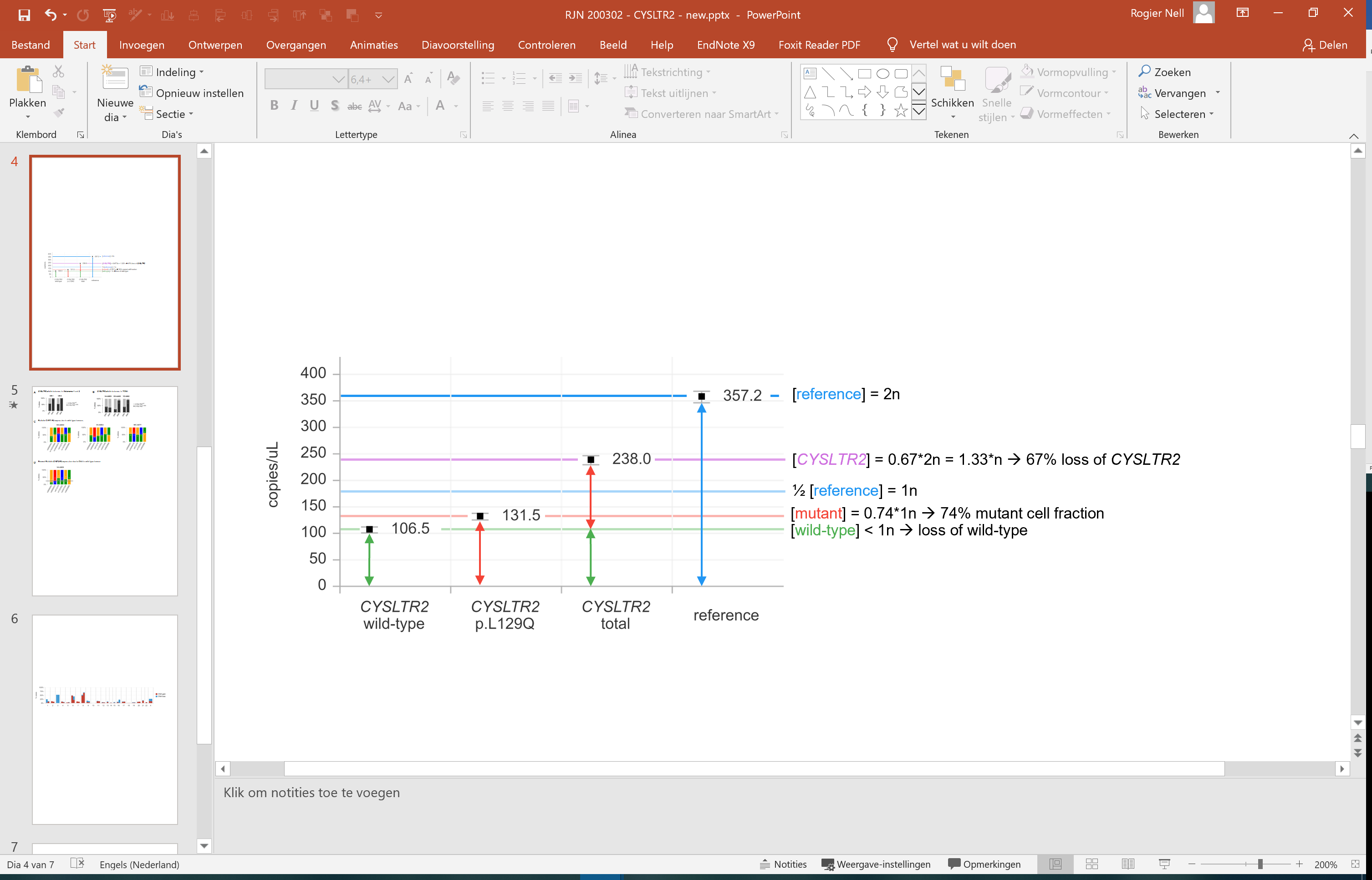


As illustrated above for PUM-1A, the simultaneous quantification of [mutant], [wild-type] and [reference] allows to determine the CNV of (total) *CYSLTR2* (i.e. chromosome 13q):

$$\text{}$$

$$\text{CNV}=2\cdot\frac{\left[ \text{CYSLTR2} \right]}{\left[ \text{reference} \right]}=2\cdot\frac{\left[ \text{wild-type + mutant} \right]}{\left[ \text{reference} \right]}$$

Moreover, the mutant cell fractions can now be calculated:

$$\mathrm{MCF}_{in case of a loss of wild-type}=2\cdot\frac{\left[ \text{mutant} \right]}{\left[ \text{reference} \right]}=CNV\cdot MAF$$

$$\mathrm{MCF}_{in case of a gain of mutant}=2-2\cdot\frac{\left[ \text{wild-type} \right]}{\left[ \text{reference} \right]}=2-\left( CNV*\left( 1-MAF \right) \right)$$

Note that these formulas also allow to determine the MCF when the CNV and MAF are determined in separate, non-multiplex experiments.

Concerning the CNV of chromosome 13q, alterations were confirmed by the allelic imbalance of heterozygous common single nucleotide polymorphism rs2057413 in *SETDB2*, located ~776kb from *CYSLTR2* (<https://www.genecards.org>):


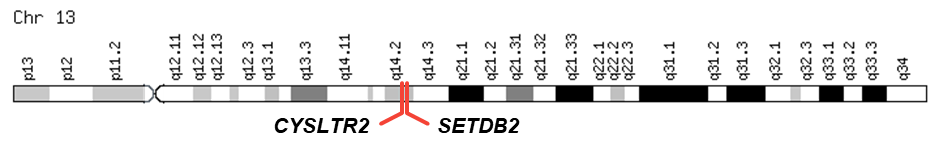


Assuming one of the alleles/variants remains stable, the SNP-based CNV was calculated as follows:

$$CNV=1+\frac{[{var}_{unstable}]}{[{var}_{stable}]}$$

Although the same result obtained using the classic (target/reference) and SNP-based CNV approach (exemplified below for PUM-1A), the SNP-based assay is a single-amplicon assay that can be used to prevent biases due to degradation of FFPE-based input material (i.e. PUM-1B and PUM-2B).


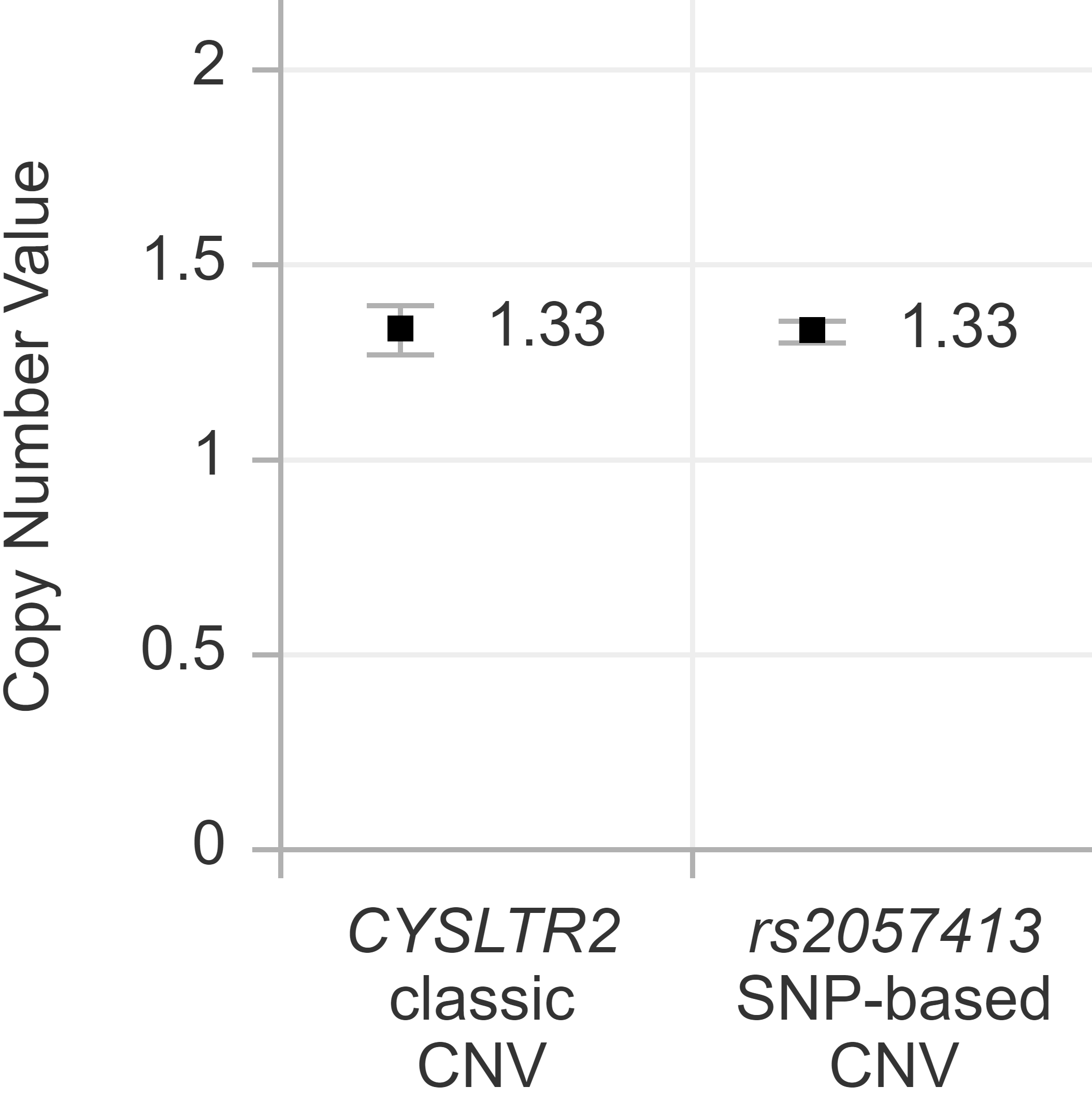


Similarly, a SNP-based CNV for chromosome 3p (rs6617 in *SPCS1*) was used, assuming the variant with the highest concentration is the stable allele.

In all cases, a CNV significantly lower than 2 was interpreted as being the sum of normal cells (CNV=2) and cells with loss (CNV=1). Consequently, the fraction cells with a loss was determined as follows:

$$\% cells with loss=\left( 2-CNV \right)\cdot100\%$$

Likewise, a CNV significantly higher than 2 was interpreted as being the sum of normal cells (CNV=2) and cells with gain (CNV=3). Consequently, the fraction cells with a gain was determined as follows:

$$\% cells with gain=\left( CNV-2 \right)\cdot100\%$$

Finally, we compared, in each tumour sample analysed, the fractions cells being *CYSLTR2* mutant with the fractions cells with a loss of chromosome 13q and 3p (PUM-1) or a gain of chromosome 13q (PUM-2):


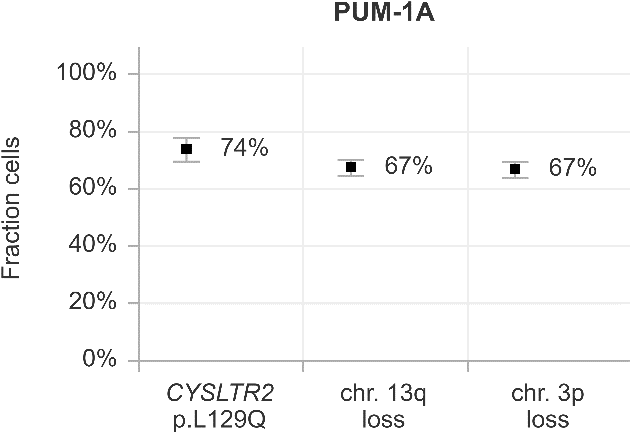

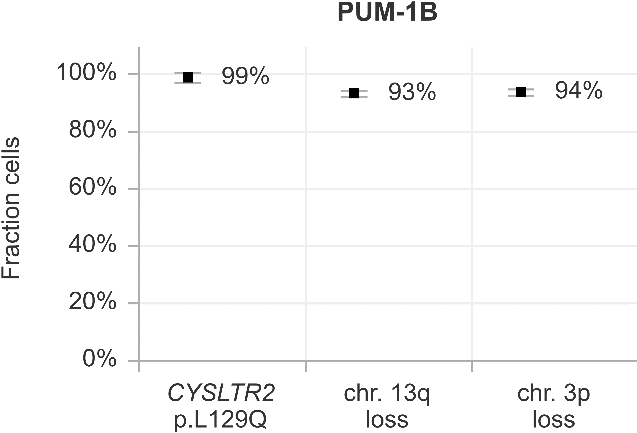


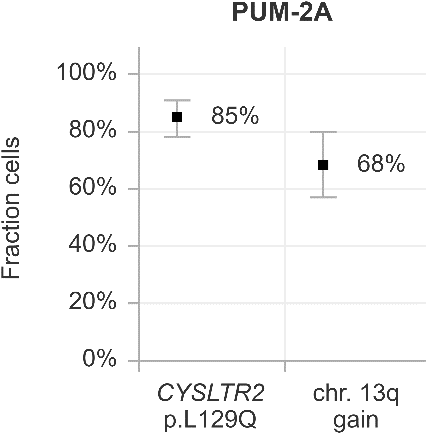

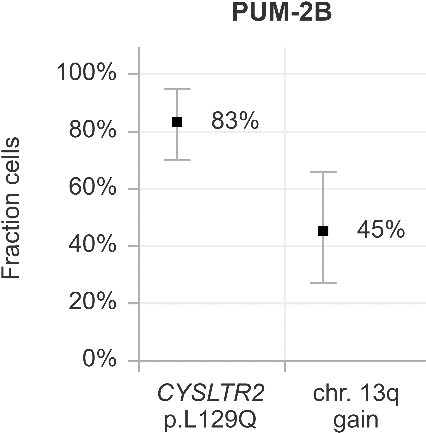


When visually comparing the calculated cell fractions, the chromosome 13q and 3p alterations were lower abundant than the *CYSLTR2* mutation with the confidence intervals typically not overlapping, suggesting a significant difference. This was confirmed in all cases (i.e. 74% versus ~67% in PUM-1A, 99% versus ~93% in PUM-1B, 85% versus 68% in PUM-2A, and 83% versus 45% in PUM-2B) by statistically comparing the obtained results (using Fieller’s theorem, p<0.05) (6).

**References for Supplementary Data 1**

1. de Lange MJ, van Pelt SI, Versluis M, Jordanova ES, Kroes WG, Ruivenkamp C, et al. Heterogeneity revealed by integrated genomic analysis uncovers a molecular switch in malignant uveal melanoma. Oncotarget. 2015;6(35):37824-35.

2. Versluis M, de Lange MJ, van Pelt SI, Ruivenkamp CA, Kroes WG, Cao J, et al. Digital PCR validates 8q dosage as prognostic tool in uveal melanoma. PLoS One. 2015;10(3):e0116371.

3. Zoutman WH, Nell RJ, van der Velden PA. Usage of Droplet Digital PCR (ddPCR) Assays for T Cell Quantification in Cancer. Methods Mol Biol. 2019;1884:1-14.

4. Robertson AG, Shih J, Yau C, Gibb EA, Oba J, Mungall KL, et al. Integrative Analysis Identifies Four Molecular and Clinical Subsets in Uveal Melanoma. Cancer Cell. 2017;32(2):204-20 e15.

5. Whale AS, Huggett JF, Tzonev S. Fundamentals of multiplexing with digital PCR. Biomol Detect Quantif. 2016;10:15-23.

6. Dube S, Qin J, Ramakrishnan R. Mathematical analysis of copy number variation in a DNA sample using digital PCR on a nanofluidic device. PLoS One. 2008;3(8):e2876.

## Supplementary Figure 1

*CYSLTR2* p.L129Q mutation detected by Sanger sequencing of choroidal nevus 12B (**A**), compared to the reference sequence from a healthy control (**B**).


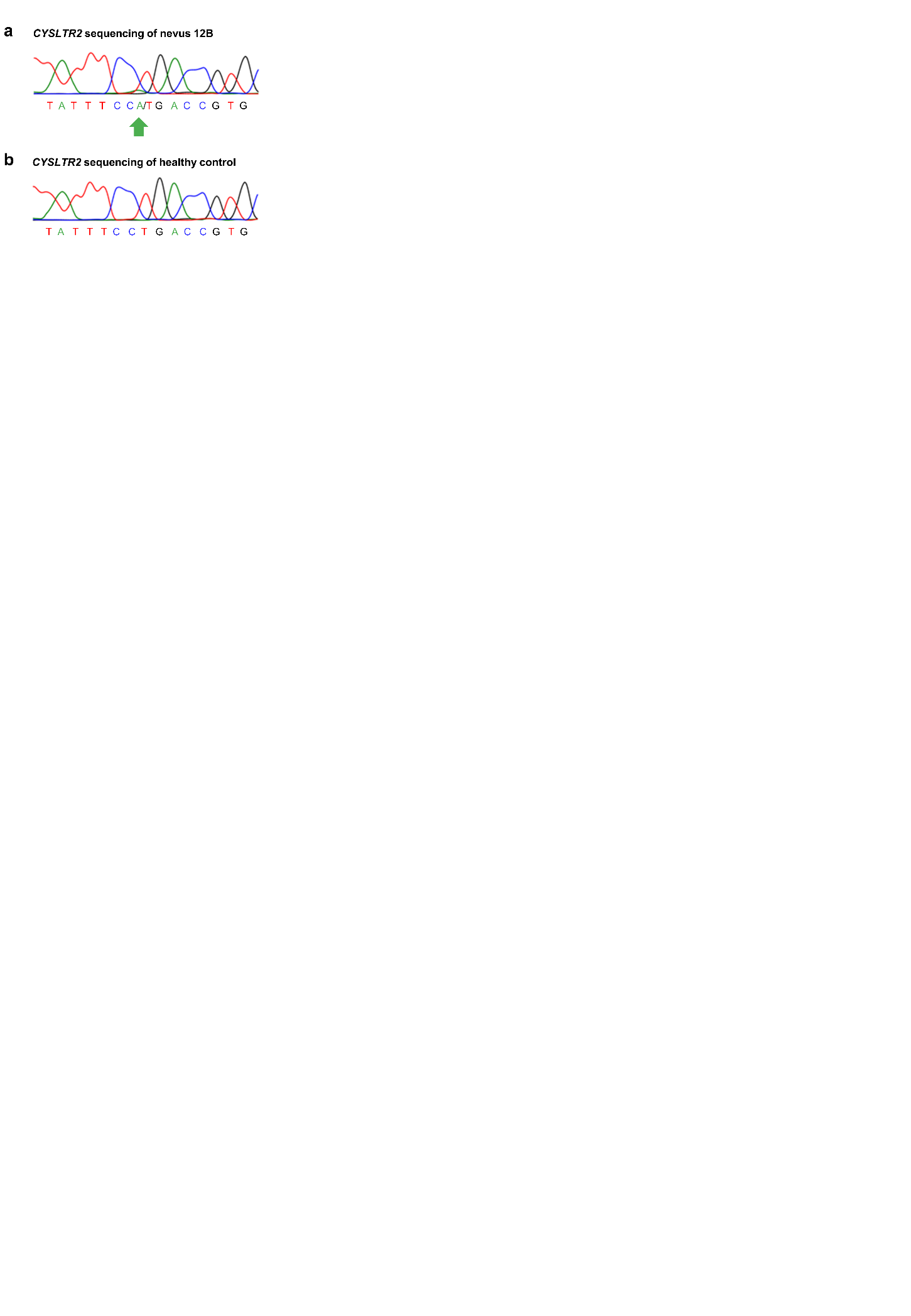


## Supplementary Figure 2

SNP-array copy number profile and B-allele fractions of *CYSLTR2* mutant melanoma TCGA-V4-A9ED (**A**), TCGA-VD-AA8O (**B**) and TCGA-YZ-A982 (**C**).


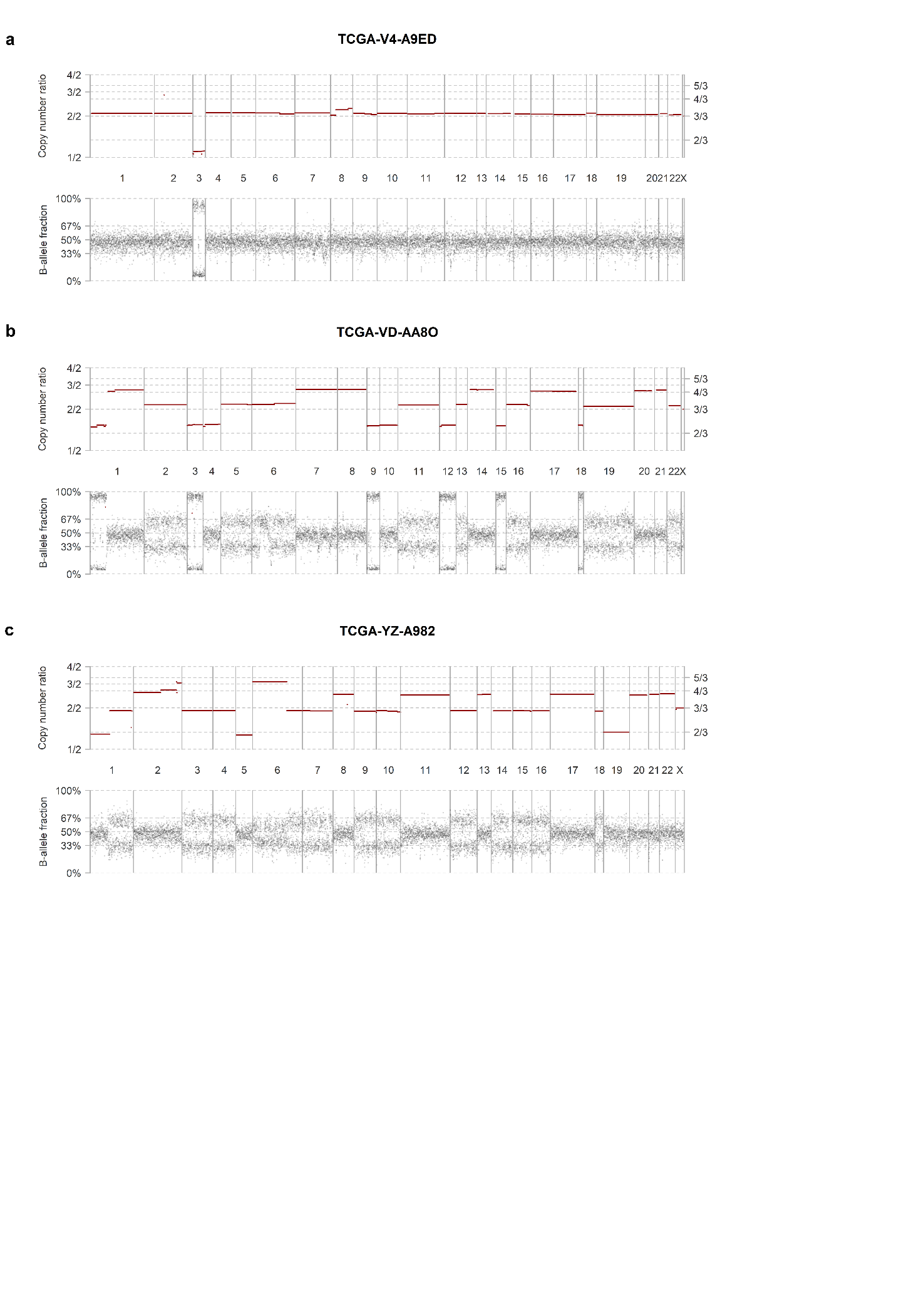


## Supplementary Figure 3

Distribution of arm-level copy number alterations in 77 *CYSLTR2* wild-type uveal melanomas from the TCGA cohort. Alterations involving chromosome 13q (*CYSLTR2* locus) are uncommon in *CYSLTR2* wild-type uveal melanomas.


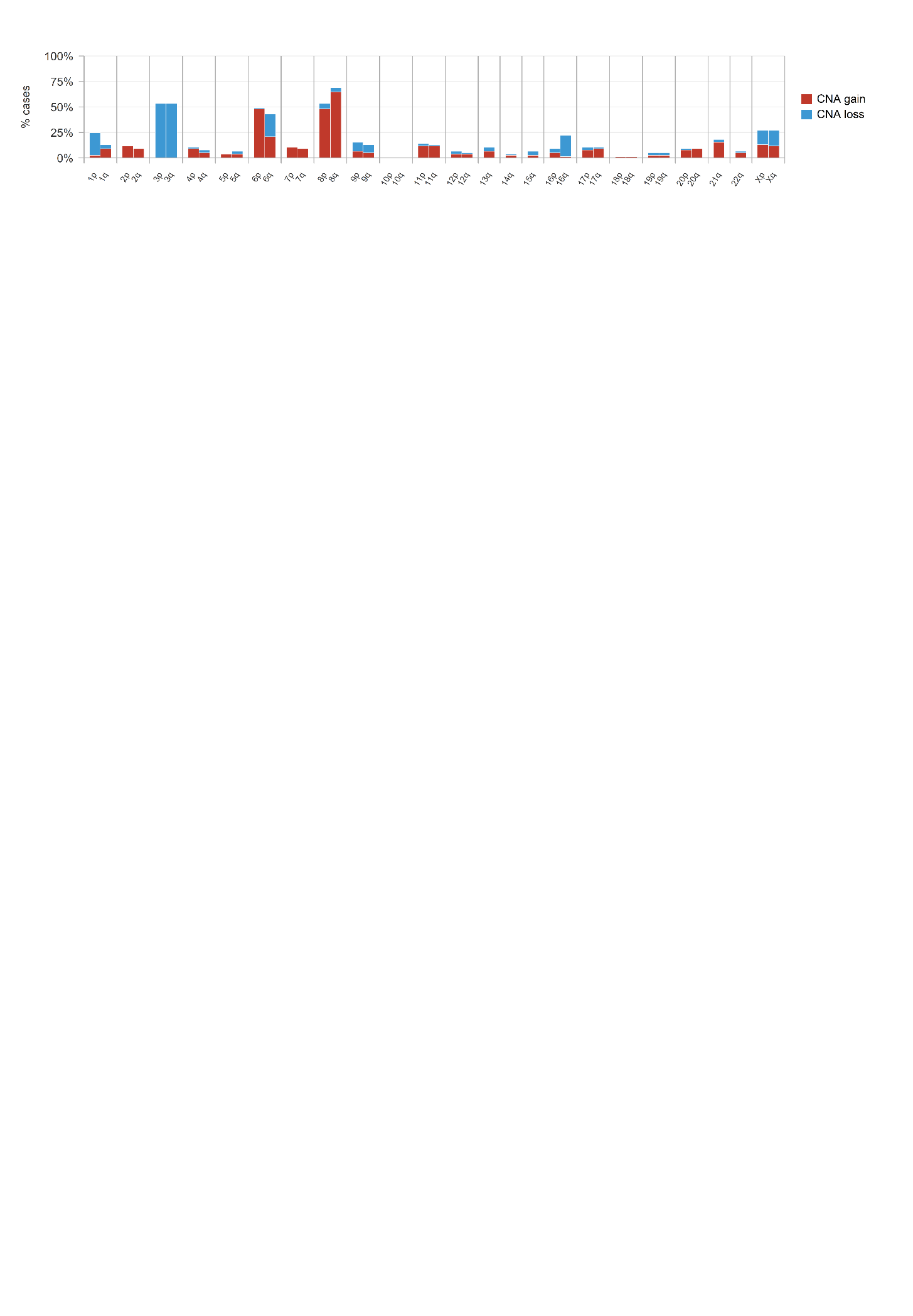


## Supplementary Figure 4

Origin of *CYSLTR2* expression and characterization of the immune microenvironment in uveal melanoma. Single cell RNA expression analysis of the six tumours with >2% *CYSLTR2*^+^ cells: primary uveal melanomas UMM059 (**A**), UMM066 (**B**), UMM064 (**C**), UMM063 (**D**) and UMM069 (**E**), and metastatic uveal melanoma UMM041L (**F**). tSNE plots per tumour are presented, with expression levels per cell of marker genes *CYSLTR2* (leukotriene receptor), *MLANA* (melanoma cells), *CD14* (monocytes/macrophages), *CD3D* (T cells), *ALOX5AP* and *ALOX5* (leukotriene synthesis), *MITF* and *TYR* (pigmentation) and *ETV5* and *DUSP4* (MAPK activation). In all tumours *CYSLTR2* was expressed in a subpopulation of melanoma cells that presented with high expression of genes involved in pigmentation. In UMM066, UMM063, UMM069 and UMM041L, *CYSLTR2* expressing cells also showed expression of the MAPK activation markers. In addition to these melanoma cells, *DUSP4* was highly expressed in T cells. Enzymes catalysing the synthesis of leukotrienes are expressed by tumour-associated immune cells, both T cells and monocytes/macrophages (e.g. in UMM063).


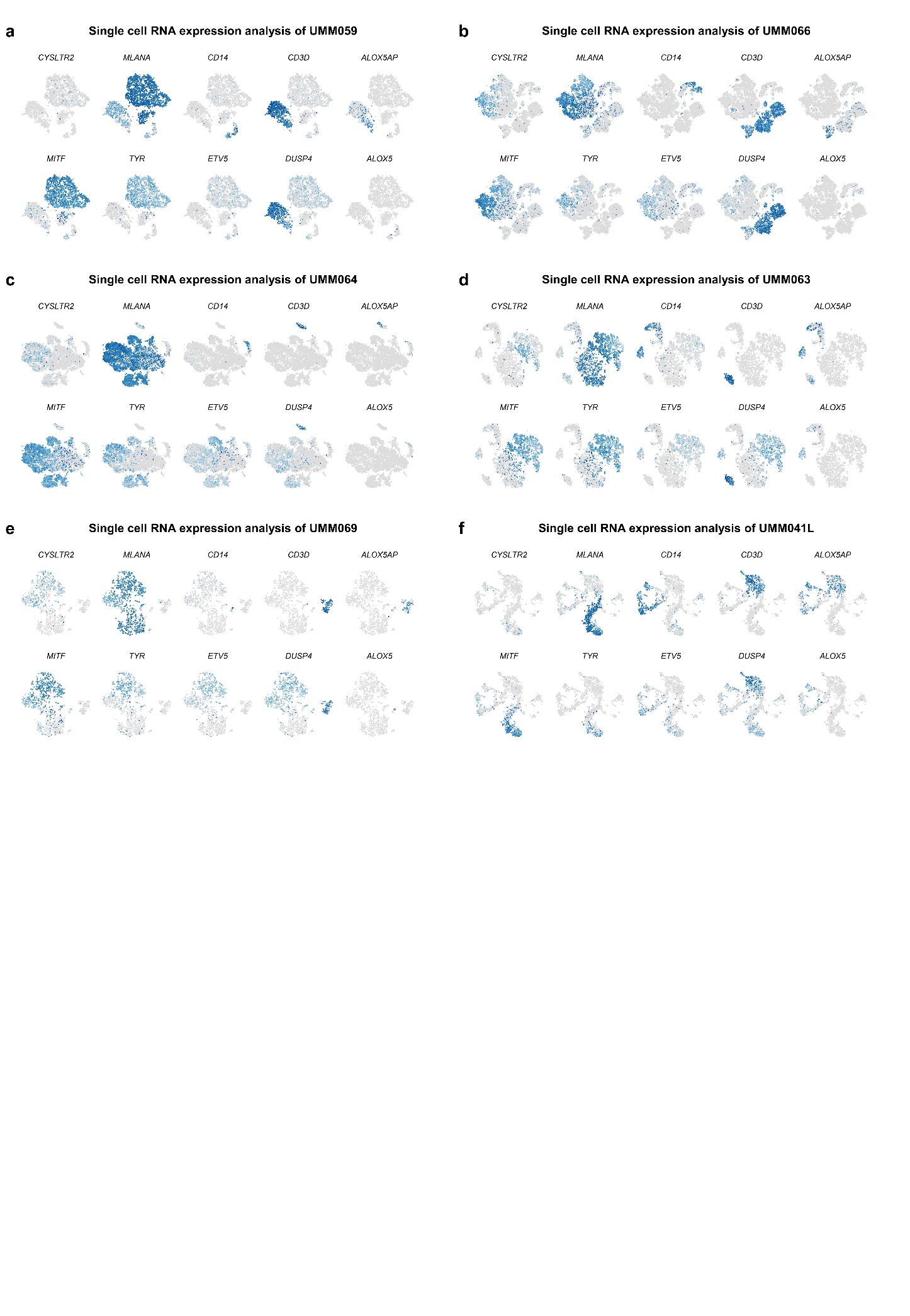

Supplement: Supplementary file 1 — Additional file 1: Supplementary Table 1. Context sequences, PCR annealing temperatures and supplier information for all assays used. Supplementary Data 1. Methodical overview of all digital PCR experimental setups. Supplementary Figure 1. CYSLTR2 p.L129Q mutation detected by Sanger sequencing of choroidal nevus 12B compared to the reference sequence from a healthy control. Supplementary Figure 2. SNP-array copy number profile and B-allele fractions of the CYSLTR2 mutant melanomas from the TCGA cohort. Supplementary Figure 3. Distribution of arm-level copy number alterations in 77 CYSLTR2 wild-type uveal melanomas from the TCGA cohort. Supplementary Figure 4. Origin of CYSLTR2 expression and characterisation of the immune microenvironment in uveal melanoma based on the single cell RNA expression analysis of the six tumours with > 2% CYSLTR2+ cells. [file 12885_2021_7865_MOESM1_ESM.docx]
